# The epidemiology and outcomes of hospitalized drowning in Thai children: a national data analysis 2015–2019

**DOI:** 10.1186/s13049-024-01270-6

**Published:** 2024-09-30

**Authors:** Sirapoom Niamsanit, Rattapon Uppala, Phanthila Sitthikarnkha, Leelawadee Techasatian, Suchaorn Saengnipanthkul, Kaewjai Thepsuthammarat, Sumitr Sutra

**Affiliations:** 1 Department of Pediatrics, Faculty of Medicine, Khon Kaen University, 123/2000 Mitraparp Rd, Muang, Khon Kaen, 40002 Thailand; 2 Clinical Epidemiology unit, Faculty of Medicine, Khon Kaen University, Khon Kaen, Thailand

**Keywords:** Drowning, Epidemiology, Burden, Children, Mortality

## Abstract

**Background:**

Drowning remains a common cause of death among children. However, the epidemiology and impact of drowning in Thailand was underexplored. This study aimed to analyze the epidemiology and clinical outcomes of pediatric drowning in Thailand and to determine the factors associated with the need for intubation and mortality.

**Methods:**

Data derived from the Thai healthcare delivery system for the period between 2015 and 2019 were used to examine the monthly admissions, mortality rates, length of hospital stay, and the number of patients who received endotracheal intubation. Multivariate logistic regression analysis was employed to identify the risk factors associated with the need for intubation and mortality.

**Results:**

Of the 4,911, 58.8% were under six years old, 63.5% were male, and 31.2% were from the Northeastern region. The majority drowned during April, which is the summer season in Thailand. Among these patients, 28.8% required intubation, with the highest proportion found in the 6-<12 years age group (35.9%). The independent risk factors for intubation were metabolic acidosis (adjusted odd ratio [aOR] 9.74; 95% confidence interval [CI] 7.14–13.29; *p* < 0.001) and pulmonary edema (aOR 5.82; 95%CI 3.92–8.65; *p* < 0.001). The overall mortality rate due to drowning was 12.6%. Factors significantly associated with mortality included in-hospital cardiac arrest (aOR 4.43; 95%CI 2.78–7.06; *p* < 0.001), and the presence of drowning-related complications, particularly renal failure (aOR 7.13; 95%CI 3.93–12.94; *p* < 0.001).

**Conclusion:**

Drowning admissions and mortality were highest among male children under six years old, occurring mainly during the summer season. Significant factors associated with intubation requirement included metabolic acidosis and pulmonary edema. The mortality was significantly associated with in-hospital cardiac arrest and drowning-related complications, particularly renal failure.

**Trial registration:**

This is an observational study, does not include any intervention, and has therefore not been registered.

**Supplementary Information:**

The online version contains supplementary material available at 10.1186/s13049-024-01270-6.

## Introduction

Drowning remains a significant public health concern worldwide, especially among pediatric populations. According to the World Health Organization, the global annual death toll from drowning in 2014 was 372,000. The majority of these deaths, 91%, occurred in low-income and middle-income countries [1]. Additionally, drowning claimed the lives of 140,219 children under the age of 15 each year, making it the third most common cause of death, following AIDS and meningitis [1]. Unintentional death from drowning decreased by 44.5% between 1990 and 2017 globally. Nevertheless, the fatal drowning rate was still high in Africa and Asia, including Thailand, with mortality rates in 2017 exceeding the global average of 4.0 per 100,000 population [2]. Past research suggested that drowning occurs more frequently in children than adults, especially among males and those living in rural environments [3].

Data from child surveys in 2000–2005 by the United Nations Children’s Fund and The Alliance for Safe Children found that drowning was a significant fatal injury in Asia, accounting for 30 per 100,000, with the most common age group being 1–4 years [4]. Therefore, preventive strategies for drowning should focus on children; however, few studies have been conducted in Thailand. Understanding the specific epidemiological factors contributing to high drowning rates could guide interventions in high-risk regions, enhancing global child health outcomes. Additionally, there has been no survey on the length of stay or the requirement for endotracheal intubation.

The primary objective of this study was to examine the epidemiology and clinical outcomes of pediatric drowning based on the Thai healthcare delivery system from 2015 to 2019. The secondary objective was to investigate the factors associated with intubation and mortality in pediatric drowning cases.

## Materials and methods

### Study setting

This study was conducted using data from the Thai healthcare delivery system, which included various levels of hospitals: primary, secondary, tertiary, and private. Primary hospitals provided basic healthcare services and were typically the first point of contact for patients. Secondary hospitals offered more specialized services and had a greater capacity for patient care. Tertiary hospitals were advanced healthcare facilities equipped with comprehensive specialized services and intensive care units. Private hospitals varied significantly in terms of resources and capabilities; some had ICU care and specialist resources comparable to tertiary care, while others had facilities similar to secondary care. Some private hospitals in Thailand were part of the National Health Security Office (NHSO) system. All hospitals within the NHSO system were included in the study to allow for a thorough analysis of healthcare services across various hospital types and levels within the country. Data that was obtained from the NHSO underwent a data auditorial system by the government prior to reimbursement to each hospital.

In cases of drowning, the nearest hospital sent an ambulance to provide prehospital care and emergency treatment upon arrival at the hospital. Patient treatment data were recorded in the NHSO system from the treating hospitals.

### Study design and participants

An observational study was utilizing data from the NHSO focused on hospitalized pediatric patients under 18 years old who were admitted due to drowning in Thailand from January 2015 to December 2019. The data of children hospitalized due to drowning were extracted using International Statistical Classification of Diseases and Related Health Problems, 10th Revision, Thai Modification (ICD-10-TM) codes such as T751 (Unspecified effects of drowning and nonfatal submersion), V90 (Drowning and submersion due to accident to watercraft), V92 (Drowning and submersion due to accident on board watercraft, without accident to watercraft), W65-74 (Accidental drowning and submersion), X92 (Assault by drowning and submersion) and Y21 (Unspecified drowning and submersion).

The inclusion criteria for this study were pediatric patients under 18 years old who were hospitalized due to drowning incidents during the study period. Exclusion criteria included patients who were dead at the scene and did not reach the hospital, or patients whose data were not recorded in the NHSO system. For the purpose of this study, admission was defined as patients who arrived at the hospital and had their data recorded in the NHSO system, regardless of whether they were admitted to the wards or not.

### Data collections

Data were collected on the age, gender, month and year of admission, hospital level, hospital region, complications, hospital length of stay (LOS), and discharge status of the patients. The data were categorized into three age groups: under 6 years, 6 to under 12 years, and 12 to under 18 years. Additionally, drowning prevalence was gathered based on gender, region, season, and hospital-level differences.

Clinical outcomes of hospitalized children due to drowning in this study included intubation requirement and hospital mortality. ICD-10-TM codes were used to define the complications including pneumonia (J189, J690, J698), acute respiratory failure (J960), acute respiratory distress syndrome (J80), pulmonary edema (J81), hypokalemia (E876), anoxic brain damage (G931), acute renal failure (N179), acidosis (E872), hyponatremia (E871), hyperglycemia (R739), and cardiac arrest with successful resuscitation (I46.0). For cardiac arrest with successful resuscitation (I46.0), we only include successfully resuscitated patients; patient death was not included. The number of patients requiring endotracheal intubation was defined using the 9th Revision, Thai Modification (ICD-9-TM) of 9671, 9672, and 9604 (continuous invasive mechanical ventilation and insert endotracheal tube). The mortality rate was calculated based on the projected national population per 100,000 people-year and stratified by age group.

### Statistical analyses

All data were analyzed using STATA software version 10.0. The qualitative data were shown as a percentage. The quantitative data were displayed as mean with standard deviation (SD) or median with interquartile range (IQR) depending on the data distribution. To ensure that the sample size was adequate for identifying significant differences in the factors associated with intubation and mortality in pediatric drowning cases, the sample size was determined using the method outlined by Hsieh et al. (1998) [5]. The sample size obtained from the database exceeded the calculation’s requirement. Univariate and backward stepwise multivariable logistic regression analysis was used to identify factors associated with endotracheal intubation requirement and mortality by estimating adjusted odds ratio (aOR) and their 95% confidence interval (CI). The factors with a *p*-value < 0.2 in the univariate analysis were entered into the model to identify the independent factors associated with intubation and mortality. *P*-values < 0.05 were considered statistically significant.

### Ethics approval

This study was reviewed and approved by the institutional review board of the Khon Kaen University Human Research Ethics Committee (#HE641154). The consent form was waived due to anonymized data. The STROBE (Strengthening the Reporting of Observational studies in Epidemiology) reporting guideline was followed [6].

## Results

### Epidemiology of hospitalized due to drowning in Thai children

**Fig. 1:**
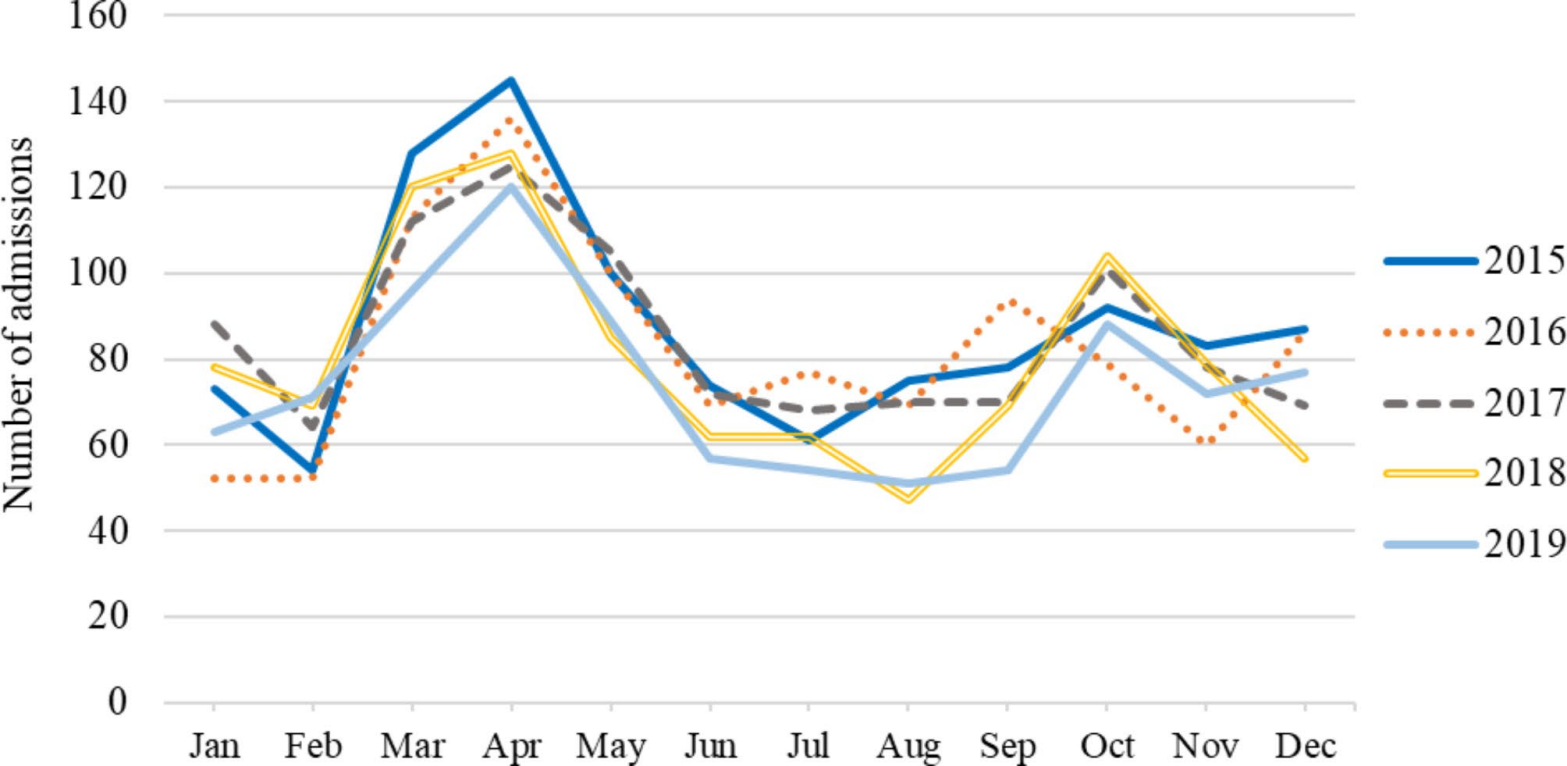
Number of admissions among children due to drowning in Thailand during 2015–2019

A total of 4,911 children who were hospitalized due to drowning were included in the study. Of them, 2,888 (58.8%) were younger than 6 years old and 3,120 (63.5%) were male. The majority (58.3%) of pediatric drowning patients were seen at secondary hospitals (Table 1). The northeast region had the highest rate of drowning (31.2%), followed by the central (30.8%) and southern (22.1%), respectively. April was the month with the highest number of drownings, followed by March and May (Fig. 1). The median length of hospital stay was 2 days (IQR 1–4).

**Table 1:** Demographic data of hospitalized children due to drowning in Thailand during 2015–2019

Factors	*n* (%)
**Year**	
2015	1,050 (21.4)
2016	987 (20.1)
2017	1,022 (20.8)
2018	960 (19.5)
2019	892 (18.2)
**Sex**	
1) Male	3,120 (63.5)
2) Female	1,791 (36.5)
**Age group**	
1) < 6y	2,888 (58.8)
2) 6-<12y	1,255 (25.6)
3) 12-<18y	768 (15.6)
**Region**	
1) Central	1,512 (30.8)
2) Bangkok	233 (4.7)
3) Northeast	1,531 (31.2)
4) North	552 (11.2)
5) South	1,083 (22.1)
**Hospital level**	
1) Primary	274 (5.6)
2) Secondary level	2,861 (58.3)
3) Tertiary	1,647 (33.5)
4) Private	129 (2.6)
**Length of Stay (Days)**	
Median (IQR)	2 (1–4)
**Discharge status**	
Alive	4,291 (87.4)
Death	620 (12.6)
**Intubation**	
Yes	1,415 (28.8)
No	3,496 (71.2)

### Prevalence and factors associated with intubation in hospitalized children due to drowning

**Fig. 2:**
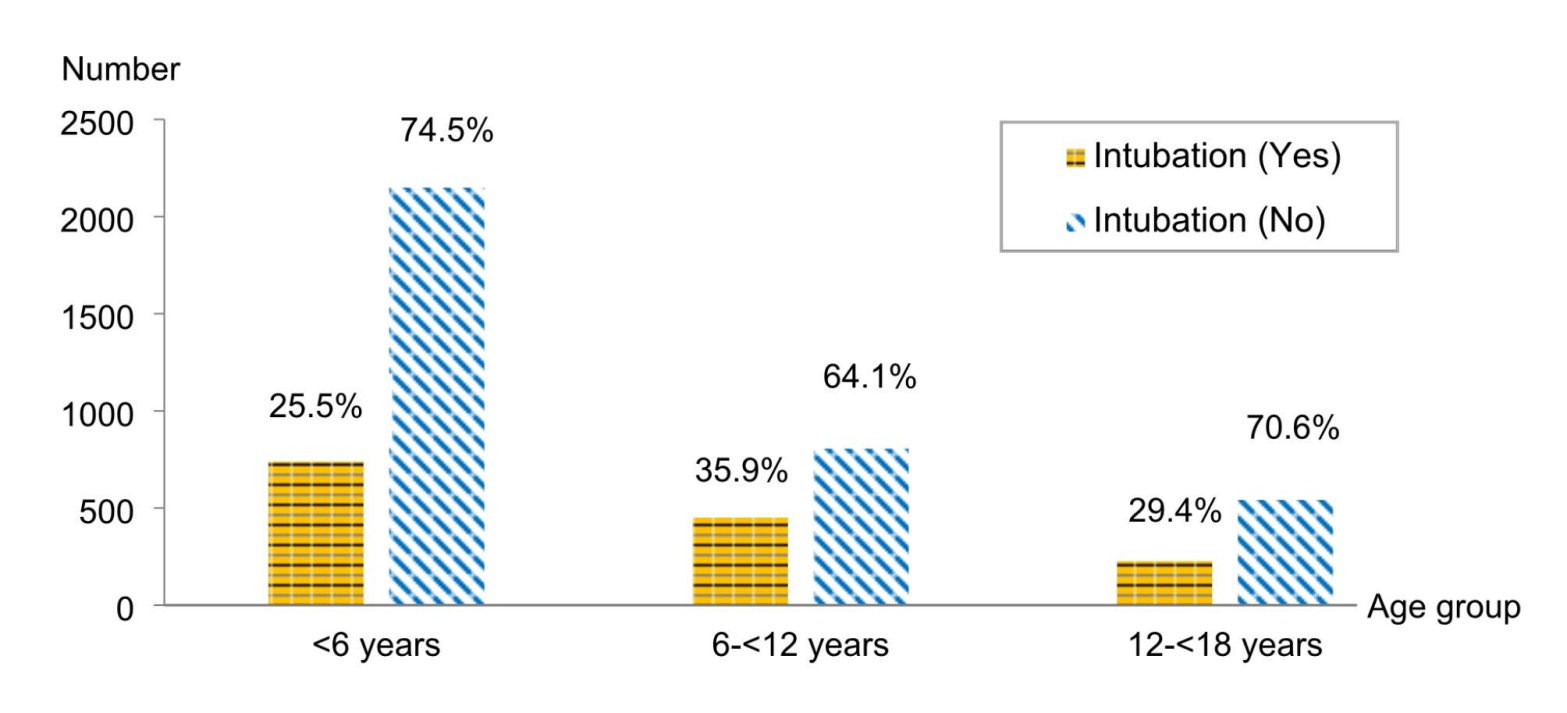
Number of intubations among hospitalized children due to drowning in Thailand during 2015–2019 stratified by age group

During the study, we found that the median length of hospital stay was 2 (IQR 1–4) days. Out of all drowned children, 1,415 (28.8%) required endotracheal intubation. The intubation rate varied across different age groups. Children 6 to under 12 years had the highest intubation rate (450 out of 1,255, 35.8%), children under 6 years of age had an intubation rate of 25.5% (739 out of 2,888), and those between 12 to less than 18 years of age had an intubation rate of 29.4% (226 out of 768) as shown in Fig. 2.

Children aged 6 to under 12 and 12 to under 18 years were associated with intubation (aOR 1.5; 95% CI 1.20–1.89; *p* < 0.001 and aOR 1.52; 95% CI 1.15–2.01; *p* < 0.001, respectively) compared to age under 6 years. Geographically, we observed the highest likelihood of intubation among patients from the northeastern region (aOR 1.5; 95%CI 1.24–1.82; *p* < 0.001). Moreover, children admitted to tertiary-level hospitals exhibited the greatest association of intubation (aOR 9.55; 95%CI 6.02–15.15; *p* < 0.001). The analysis also noted a significant association between intubation rate and drowning-related complications including pneumonitis, hypokalemia, metabolic acidosis, and pulmonary edema. Among these, metabolic acidosis (aOR 9.74; 95%CI 7.14–13.29; *p* < 0.001) and pulmonary edema (aOR 5.82; 95%CI 3.92–8.65; *p* < 0.001) represented the most significant risk factors for intubation. (Table 2)

**Table 2:** Factors associated with intubation among hospitalized children due to drowning in Thailand during 2015–2019

Factors	Intubation		Crude OR	95%CI	*p*-value	Adjusted OR	95%CI	*p*-value
	Yes (*n* = 1,415)	No (*n* = 3,496)						
**Age group (years)**					< 0.001			< 0.001
<6	739 (52.2)	2,149 (61.5)	1	-		1	-	
6-<12	450 (31.8)	805 (23.0)	1.65	1.42–1.90		1.5	1.20–1.89	
12-<18	226 (16.0)	542 (15.5)	1.22	1.02–1.46		1.52	1.15–2.01	
**Region**					0.002			< 0.001
Central	383 (27.1)	1,129 (32.3)	1	-		1	-	
Bangkok	67 (4.7)	166 (4.8)	1.19	0.87–1.62		1.28	0.86–1.93	
Northeast	492 (34.8)	1,039 (29.7)	1.40	1.19–1.64		1.50	1.24–1.82	
North	163 (11.5)	389 (11.1)	1.24	0.99–1.54		1.52	1.17–1.99	
South	310 (21.9)	773 (22.1)	1.18	0.99–1.41		1.21	0.97–1.50	
**Hospital level**					< 0.001			< 0.001
Primary	26 (1.8)	248 (7.1)	1	-		1	-	
Secondary	517 (36.6)	2,344 (67.0)	2.01	1.34–3.01		2.31	1.46–3.67	
Tertiary	858 (60.6)	789 (22.6)	9.73	6.50-14.56		9.55	6.02–15.15	
Private	14 (1.0)	115 (3.3)	1.21	0.63–2.35		1.38	0.60–3.15	
**Co-diagnosis**								
Pneumonitis	493 (34.8)	713 (20.4)	2.1	1.84–2.41	< 0.001	1.87	1.58–2.21	< 0.001
Hypokalemia	423 (29.9)	238 (6.8)	5.73	4.83–6.81	< 0.001	4.14	3.36–5.10	< 0.001
Metabolic acidosis	320 (22.6)	78 (2.2)	12.42	9.65–15.98	< 0.001	9.74	7.14–13.29	< 0.001
Pulmonary edema	147 (10.4)	56 (1.6)	6.61	4.87–8.98	< 0.001	5.82	3.92–8.65	< 0.001

### Mortality rate and factors associated with mortality in hospitalized children due to drowning

**Fig. 3:**
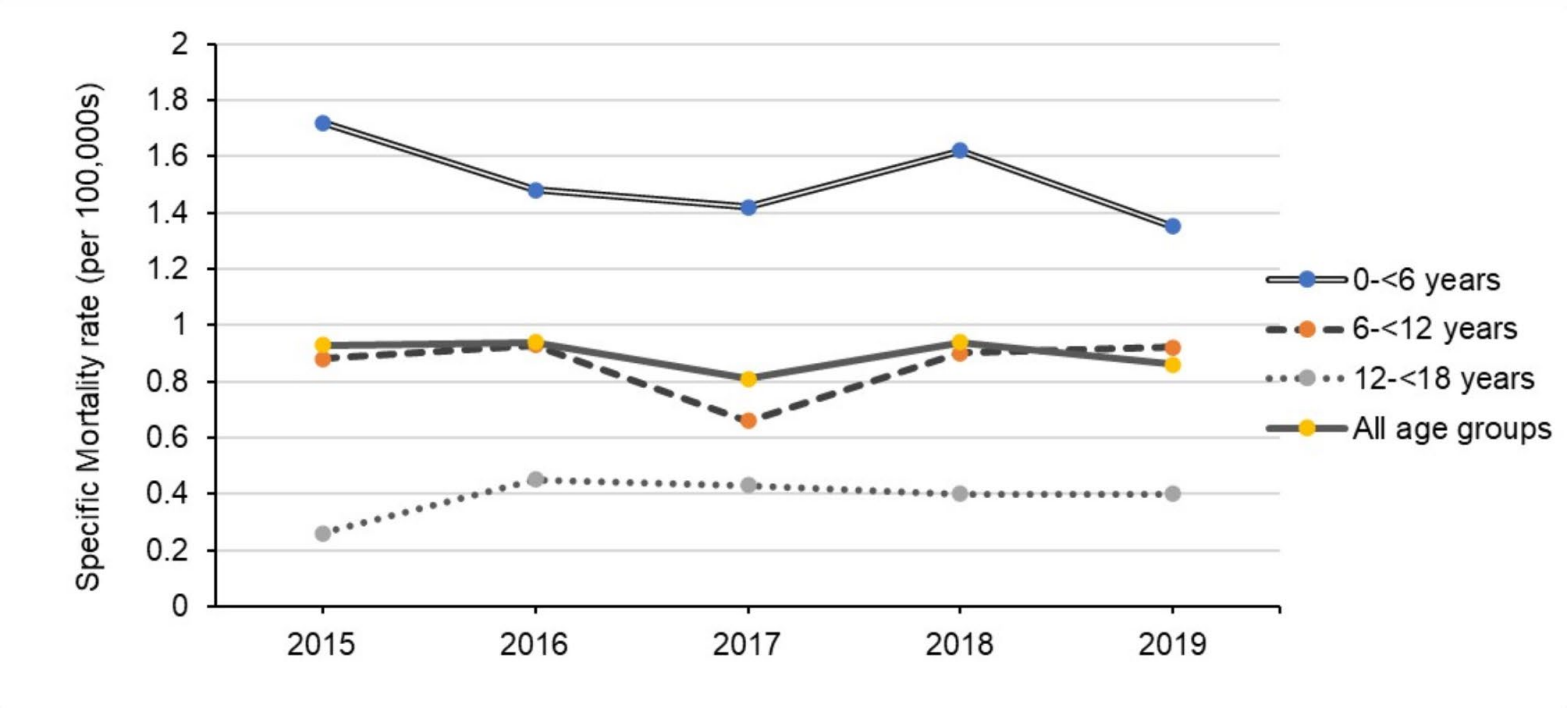
Mortality rate of hospitalized children due to drowning in Thailand during 2015–2019

The number of deaths in hospitalized children due to drowning during the study period was 620, accounting for 12.6%. The mortality rate ranged from 1.35 to 1.72 per 100,000 person-year during 2015–2019. Male children had twice the rate of deaths compared to females. This study also found that children under the age of 6 were at the highest risk of drowning-related deaths. The years 2016 and 2018 witnessed the greatest rates of drowning mortality (Fig. 3).

Drowning complications, especially acute renal failure (aOR 7.13; 95%CI 3.93–12.94; *p* < 0.001), in-hospital cardiac arrest with successful resuscitation (aOR 4.43; 95%CI 2.78–7.06; *p* < 0.001), anoxic brain damage (aOR 2.41; 95%CI 1.66–3.48; *p* < 0.001), acute respiratory distress syndrome (aOR 2.34; 95%CI 1.60–3.42; *p* < 0.001), hyperglycemia (aOR 2.78; 95%CI 1.70–4.55; *p* < 0.001) and metabolic acidosis (aOR 4.15; 95%CI 2.99–5.75; *p* < 0.001) emerged as the most significant associated factors for mortality of hospitalized children due to drowning. On the other hand, pneumonitis (aOR 0.22; 95%CI 0.16–0.30; *p* < 0.001) and hypokalemia (aOR 0.39; 95%CI 0.28–0.53; *p* < 0.001) were found to be significant protective factors against mortality. (Table 3)

**Table 3:** Factors associated with mortality among hospitalized children due to drowning in Thailand during 2015–2019

Factors	Death		Crude OR	95%CI	*p*-value	Adjusted OR	95%CI	*p*-value
	Yes (*n* = 565)	No (*n* = 850)						
**Age group (years)**					0.412			
<6	302 (53.5)	437 (51.4)	1	-				
6-<12	181 (32.0)	269 (31.7)	0.97	0.77–1.24				
12-<18	82 (14.5)	144 (16.9)	0.81	0.60–1.11				
**Region**					< 0.001			< 0.001
Central	177 (31.3)	206 (24.2)	1	-		1	-	
Bangkok	23 (4.1)	44 (5.2)	0.64	0.38–1.10		0.56	0.28–1.13	
Northeast	206 (36.5)	286 (33.7)	0.85	0.65–1.11		0.74	0.53–1.03	
North	65 (11.5)	98 (11.5)	0.79	0.54–1.15		0.82	0.53–1.29	
South	94 (16.6)	216 (25.4)	0.51	0.37–0.70		0.35	0.24–0.52	
**Hospital level**					0.171			< 0.001
Primary	12 (2.1)	14 (1.6)	1	-		1	-	
Secondary	225 (39.8)	292 (34.4)	0.94	0.43–2.07		0.58	0.22–1.52	
Tertiary	322 (57.0)	536 (63.1)	0.78	0.21–2.89		0.42	0.09–2.03	
Private	6 (1.1)	8 (0.9)	0.74	0.34–1.61		0.31	0.12–0.80	
**Co-diagnosis**								
Pneumonitis	101 (17.9)	392 (46.1)	0.26	0.20–0.33	< 0.001	0.22	0.16–0.30	< 0.001
Hypokalemia	127 (22.5)	296 (34.8)	0.54	0.42–0.69	< 0.001	0.39	0.28–0.53	< 0.001
Metabolic acidosis	218 (38.6)	102 (12.0)	4.55	3.48–5.95	< 0.001	4.15	2.99–5.75	< 0.001
Anoxic brain damage	130 (23.0)	89 (10.5)	2.54	1.89–3.40	< 0.001	2.41	1.66–3.48	< 0.001
ARDS*	123 (21.8)	76 (8.9)	2.87	2.11–3.91	< 0.001	2.34	1.60–3.42	< 0.001
Cardiac arrest**	103 (18.2)	36 (4.2)	4.97	3.35–7.39	< 0.001	4.43	2.78–7.06	< 0.001
Acute renal failure	95 (16.8)	20 (2.4)	8.44	5.13–13.88	< 0.001	7.13	3.93–12.94	< 0.001
Hyperglycaemia	95 (16.8)	35 (4.1)	4.62	3.09–6.92	< 0.001	2.78	1.70–4.55	< 0.001

*Acute respiratory distress syndrome, **Cardiac arrest with successful resuscitation

## Discussion

According to the Thai healthcare delivery system data from 2015 to 2019, the mortality rate due to drowning was similar to that reported in Australia [7]. This comparison underscores the severity of drowning incidents in both countries and underscores the need for effective drowning prevention strategies, despite differences in geographic location, healthcare systems, and population demographics. The age group with the highest mortality rate was children under six, which aligns with prior studies [8, 9]. Younger children, often unsupervised, are more likely to drown during bathing or in pools, typically having minimal swimming skills and frequently unwitnessed events, leading to worse outcomes [10, 11]. This contrasts with adolescent drowning incidents which are commonly observed and occur primarily in natural water bodies [12, 13].

Most drowning events involved males, consistent with findings from other studies [7, 14]. This suggests that greater participation of boys in outdoor activities could be a contributing factor [9]. Drowning incidents peaked annually in April, during the summer season in Thailand, similar to the results of other study [15] Reasons for higher drowning rates in summer include hot weather attracting children to water bodies and increased alcohol consumption among adolescents, a risk factor for drowning. Geographically, most drownings occurred in the northeast region, which had the lowest average monthly income per household [16]. Furthermore, higher mortality rates were noted in this region and the central region when compared to the south region and Bangkok.

The trends in our research have remained consistent from 2001 to 2009 to 2015–2019, with the highest mortality rates consistently observed among children under six and males in low socioeconomic regions [8, 9]. This supports earlier findings of higher drowning incidence and mortality in rural areas with a lower socioeconomic status and educational levels [17–19]. Despites various policies in drowning prevention, these may not have been fully effective due to persistent socioeconomic and educational disparities in rural areas, limiting access to swimming lessons and water safety education [20]. Additionally, rural communities often face challenges like limited access to emergency medical services and lower overall awareness of drowning risks [21]. Therefore, focusing drowning prevention strategies on these persistent high-risk groups could significantly reduce the mortality rate.

Drowning can lead to serious complications such as pulmonary edema, aspiration pneumonia, and ARDS, causing acute respiratory failure and occasionally cardiac arrest. These complications are primary triggers for endotracheal intubation requirement and multiorgan failure [22]. According to our study, a significant portion of drowning patients required intubation, paralleling findings from previous studies [23]. Children aged between 6 and 18 years were more likely to require intubation, possibly due to witnessed accidents in pools or natural waters, resulting in a higher level of intervention compared to the younger age group [11–13].

Our study revealed that patients suffering from cardiac arrest and other drowning-related complications, particularly renal failure, had a high mortality rate. Consistent with previous research, children requiring advanced CPR were more likely to have had unwitnessed or prolonged submersion injury, consequently increasing the risk of fatal outcomes [13]. Patients with hemodynamic instability, renal failure, ARDS, hyperglycemia and metabolic acidosis were significantly higher risk of multiorgan dysfunction and cardiac arrest, leading to death or extended hospital stays in non-fatal cases [10, 22, 24, 25]. Early detection, immediate CPR, and bystander intervention could significantly reduce severe outcomes, including a decrease in the rate of intubation, and shorten hospital stays [26].

Pneumonitis was found to be a protective factor in our study. This contrasts with the more severe complication of ARDS, which often leads to poorer outcomes. This may be explained by the fact that the pulmonary lesion caused by pneumonitis is often temporary and localized. Consequently, these patients tend to recover much faster than patients with ARDS, and late pulmonary sequelae are uncommon [27]. Hypokalemia also emerged as a protective factor. The previous research found that nearly half of the patients who survive after drowning had hypokalemia, in contrast to hyperkalemia, which is commonly found in non-survivor patients. Hypokalemia might indicate a milder metabolic response. In contrast, hyperkalemia, often resulting from significant red cell lysis and cellular breakdown, is associated with severe injury and poor outcomes [28, 29]. Therefore, early detection and management of drowning complications before the progression of organ failure are crucial for improving survival outcomes.

Our study highlighted the need for targeted drowning prevention efforts. Based on our findings, targeted drowning prevention strategies are crucial. Since males and younger children in low socioeconomic areas were found to be at higher risk, specific measures should be implemented, especially during the summer months. These measures include providing adequate parental supervision and water hazard awareness education. Early detection and effective management of complications from drowning, such as pulmonary edema, aspiration pneumonia, and ARDS, are crucial. Prompt referrals to tertiary care facilities with intensive care specialists for close monitoring could enhance recovery outcomes and reduce mortality rates.

### Strengths and limitations

This study uses extensive national data from Thailand’s healthcare system over five years, providing a detailed analysis of pediatric drowning incidents. The use of reliable national data is a significant strength, as it allows for comprehensive insights into the trends and risk factors associated with intubation and mortality due to drowning.

However, the study has certain limitations. Its retrospective design makes it susceptible to potential coding errors, while the absence of a control group limits comparative insights. There may be underreporting of non-hospitalized drowning cases, and reliance on hospital data could distort the actual incidence rates. Additionally, the study does not differentiate between drowning types in various water bodies and lacks data on clinical drowning severity, transportation modes, and witness status, potentially affecting the applicability of specific preventive measures. The exclusive use of ICD-10 coding could result in missed cases due to miscoding. Consequently, the database contains minimal amounts of missing data. However, the classification of certain items using ICD-10 may be inaccurate. Nevertheless, our research is limited to life-threatening conditions with straightforward diagnoses, which typically have fewer miscoding in the database. Therefore, we declare this to be our limitation. Furthermore, the absence of long-term outcome measures beyond intubation and mortality limits a comprehensive understanding of the incident’s impact. These factors highlight the need for future studies to address these gaps and improve the generalizability and applicability of findings across various regions and settings.

## Conclusions

This study highlights the significant impact of pediatric drowning on morbidity and mortality in Thailand, with an overall mortality rate of 12.62% and intubation required in over a quarter of cases. The findings underscore the need for targeted prevention strategies, especially for males under six in low socioeconomic regions. Early detection, effective management of complications, and prompt referral to specialized care can improve outcomes and reduce mortality rates. Future research should focus on implementing these strategies nationwide.

## Electronic supplementary material

Below is the link to the electronic supplementary material.

Supplementary Material 1

## Data Availability

The datasets generated and/or analyzed during the current study are not publicly available, but available from the corresponding author (RU) on request.
